# Fish Burgers Fortified with Microencapsulated Sacha Inchi Oil: Effects on Technological and Sensory Properties

**DOI:** 10.3390/foods13071004

**Published:** 2024-03-26

**Authors:** Estefany Rengifo, Juan D. Rios-Mera, Patricia Huamaní, Rafael Vela-Paredes, Jessy Vásquez, Erick Saldaña, Raúl Siche, Fernando Tello

**Affiliations:** 1 Departamento de Ingeniería de Alimentos, Facultad de Industrias Alimentarias, Universidad Nacional de la Amazonía Peruana, Iquitos 16002, Peru; estefany.rengifo@unapiquitos.edu.pe (E.R.); patriciahuamanimendoza4@gmail.com (P.H.); rafael.vela.515@unapiquitos.edu.pe (R.V.-P.); jessy.vasquez@unapiquitos.edu.pe (J.V.); 2 Instituto de Investigación de Ciencia y Tecnología de Alimentos (ICTA), Universidad Nacional de Jaén, Jaén 06800, Peru; juan.rios@unj.edu.pe; 3 Sensory Analysis and Consumer Study Group, Escuela Profesional de Ingeniería Agroindustrial, Universidad Nacional de Moquegua, Moquegua 18001, Peru; esaldanav@unam.edu.pe; 4 Escuela de Ingeniería Agroindustrial, Facultad de Ciencias Agropecuarias, Universidad Nacional de Trujillo, Trujillo 13011, Peru; rsiche@unitru.edu.pe

**Keywords:** spray-drying, fish products, microparticles, sacha inchi oil, omega 3

## Abstract

The long-chain omega-3 fatty acids alpha linolenic acid (ALA), eicosapentaenoic acid (EPA), and docosahexaenoic acid (DHA) have proven health benefits, but it is not common to find them together in a processed food product. This could lead to healthier and more functional food products, which may have positive implications for consumer health and well-being. This work aimed to fortify a model burger manufactured with fillets of an Amazonian fish (boquichico, *Prochilodus nigricans*) by adding microencapsulated sacha inchi oil (*Plukenetia volubilis,* rich in ALA) (MSIO) produced by spray-drying. MSIO was incorporated into the burgers at different levels (0, 3, 4, 5, and 6%). The burgers were characterized by their proximal composition, cooking losses, texture profile, lipid oxidation, sensory profile, overall liking, and fatty acid profile. The results showed that adding MSIO up to concentrations of 5% or 6% increased the instrumental hardness, chewiness, and lipid oxidation in the burgers. However, fortifying the burgers with 3% MSIO was possible without affecting the burgers’ sensory properties and overall liking. Regarding the fatty acid profile, the burgers with 3% MSIO had a higher content of polyunsaturated fatty acids, with the ALA, EPA, and DHA types of fatty acids. Therefore, we recommend using this fortification concentration, but future studies should be carried out to improve the oxidative stability of MSIO and the burgers.

## 1. Introduction

Nowadays, consumer preferences are driven towards fast food consumption due to its time-saving convenience, which aligns with busy lifestyles [1]. Burgers are among the most popular fast food products worldwide [2]. However, they bring about serious health concerns since they contain high amounts of saturated fatty acids [3]. For this reason, numerous research endeavors have been undertaken to showcase the viability of fish burgers as a nutritious substitute, highlighting their potential health benefits [4,5,6,7]. 

The Peruvian Amazon basin has a wide diversity of fish. Among them, the boquichico (*Prochilodus nigricans*) is one of the most consumed species as it is part of the local population’s regular diet, being a good source of proteins and vitamins, among other nutrients [8]. It is mainly sold fresh and generally whole since it has many thorns. However, fresh fish are susceptible to microbial contamination and enzymatic and chemical deterioration, resulting in a decline in their sensory attributes and a shortened shelf-life. [9]. 

To solve these problems, adding value to fish products through industrialization is necessary, especially in those regions with the greatest demand, such as Loreto, the area with the largest territory in the Peruvian Amazon. Loreto was declared, in 2022, the least competitive region of Peru, whose low economic index and infrastructure level stand out [10]. Therefore, the proposal for the industrialization of Amazonian resources could improve the competitiveness of Loreto, preferably in the production of foods of high mass consumption, as is the case of burgers, in the sense of guaranteeing consumers’ acceptability.

Burgers also provide the alternative of incorporating nutrients that are typically absent or found in small amounts, such as adding n-3 polyunsaturated fatty acids (n-3 PUFAs). In this context, the use of fish fillets by themselves can provide long-chain eicosapentaenoic acid (EPA) and docosahexaenoic acid (DHA), the positive effects of which have been demonstrated on health, including anti-inflammatory, wound-healing, neuroprotective, cardioprotective, and hepatoprotective effects [11]. However, seed plant oils can also provide n-3 PUFAs because they are n-3 alpha-linolenic acid (ALA) sources. In this sense, developing foods with ALA, EPA, and DHA would be a highly nutritious alternative for consumers.

Sacha inchi (*Plukenetia volubilis*), also known as Inca peanut, is an oilseed plant native to the Amazonian region of Peru and several other countries in South America [12]. Sacha inchi oil (SIO) presents high PUFA values, mainly ALA (46–54%) and linoleic acid (33–37%), which account for approximately 80–90% of the total oil content [13]. Likewise, SIO has been recognized for its numerous health benefits, which are related to the presence of tocopherols, carotenes, phytosterols, and polyphenols [14,15]. However, the direct incorporation of oils rich in polyunsaturated fatty acids (PUFAs) in food items can negatively impact crucial technological and sensory attributes of said foods because of their high susceptibility to oxidation [16]. For this reason, microencapsulation technologies have been proposed to promote these oils’ protection and thermal stability, representing a suitable alternative for their incorporation into food products [17]. Spray-drying stands out as one of the most prevalent microencapsulation methods due to its simplicity, reproducibility, and cost-effectiveness [18]. This method generates high-quality powdered microparticles with reduced water activity, simplifying their transportation, handling, and storage. Additionally, spray-drying is particularly suitable for encapsulating materials sensitive to heat, such as PUFA-rich oils, since their drying times are usually short (a few seconds) [19]. However, conflicting findings have emerged concerning the oxidative stability of PUFAs encapsulated by means of spray-drying, due to factors such as temperature, elevated moisture levels in the drying chamber, wall materials, and payload of the core oil, among others [20]. Therefore, it is necessary to demonstrate the protective effect of spray-drying PUFAs under established encapsulation conditions, compared with non-encapsulated PUFA-rich oil.

In the preparation of burgers, it is usual to add animal fats to increase the yield and improve the product’s sensory traits. A traditional ingredient is pork back fat, but it is known to have high saturated fat and cholesterol levels. In this sense, the fortification of fish-based burgers with microencapsulated sacha inchi oil (MSIO) in this study was related to the partial replacement of pork back fat. Therefore, this work aimed to assess the effects of different levels of MSIO incorporation on the physicochemical and technological properties, oxidative stability, and sensory and fatty acid profiles of the developed fish burgers.

## 2. Materials and Methods

### 2.1. Materials

Sacha inchi oil (SIO) was obtained from Shanantina S.A. (Lamas, Tarapoto, Peru); Arabic gum (AG), inulin (Inu), and sodium erythorbate (antioxidant) were purchased from Frutarom Perú S.A. (Lima, Peru); polysorbate 80 was obtained from Insuquimica S.A.C. (Lima, Peru). The boquichico (*Prochilodus nigricans*) fish was sourced from the fish farm “Fundo Tony” in Iquitos, Peru. After filleting, the fish was vacuum-sealed and kept at a temperature of −18 °C for less than a week. The fillets presented 74.25 ± 0.34% of moisture, 18.56 ± 0.22% of protein, 4.79 ± 0.04% of fat, 1.09 ± 0.12% of ash, and 1.21 ± 0.16% of carbohydrates, which were determined with the AOAC (2012) methods described in Section 2.5. The pork back fat and spices were acquired from supermarkets in Iquitos, Peru. The oregano powder was sourced from Badía (Lima, Peru).

### 2.2. SIO Microencapsulation by Spray-Drying

MSIO was produced following the methodology described by Nogueira et al. [21], with modifications. The feed solution was prepared by solubilizing 100 g of a mixture of AG–Inu (0.854:0.146) in 1000 mL of distilled water, which was kept under constant stirring for 12 h. Then, 30 g of SIO and 1.5 g of polysorbate 80 (emulsifying agent) were incorporated and homogenized utilizing an Ultra-Turrax homogenizer (Borg Brand, Model WT-500; Wertheim, Germany) for 3 min at 18,000 rpm. Then, the emulsion was fed into a LabPlant SD-Basic spray-dryer (LabPlant, Huddersfield, UK) under the following conditions: a nozzle diameter of 1.0 mm, an inlet temperature of 163 °C, and an airflow rate of 73 m^3^/h. At the end of the spray-drying process, the resulting microparticles were stored in a refrigerator (5 to 7 °C) in vacuum-sealed aluminum foil until further analysis.

### 2.3. Encapsulation Efficiency (EE), Oxidative Stability, and Microstructure

EE was determined in triplicate by calculating the total oil (TO) and the surface oil (SO), following the methodology reported by Bae and Lee [22], using Equation (1).

(1) $$EE=\left(\frac{TO-SO}{TO}\right)\times 100$$

The microstructure analysis of the samples was performed via scanning electron microscopy (SEM). The oxidative stability of MSIO and unencapsulated sacha inchi oil (USIO) was assessed weekly for four weeks at 45 °C in a climate chamber (Climacell ECO 111, MMM Group, Munich, Germany) by determining the peroxide values according to the standard method of IDF 74A: 1991 [23]. For MSIO, the oil was previously extracted following the Bligh and Dyer [24] method. The amount of peroxides generated was quantified by referencing a standard curve for Fe^3+^, with concentrations varying from 1 to 20 µg [25,26,27].

### 2.4. Burger Manufacturing

Six treatments of burgers were applied in three independent manufacturing processes (batches): the treatment without SIO (T1)0 and the five treatments of SIO fortification at 3% USIO (T2), 3% MSIO (T3), 4% MSIO (T4), 5% MSIO (T5), and 6% MSIO (T6). In each independent process, 30 burgers per treatment were produced.

The burgers’ manufacturing, molding, and storage were carried out following the methodology described by Iman et al. [27]. Table 1 details the different formulations and ingredient concentrations used.

For the burgers’ analyses, the samples were first thawed at room temperature (25 °C) for around 15 min. The burgers were cooked for around 10 min on an electrical grill at 150 °C until the internal temperature of the fish burgers reached 75 °C; then, the texture profile, cooking losses, and sensory analyses were carried out. The samples were cooled at 25 °C for the texture profile and cooking losses analyses and at 45 °C for the sensory analysis.

### 2.5. Proximal Analysis

The methodologies described by AOAC [28] were used for the proximal composition of the raw burgers: moisture (method, 950.46), protein (method 981.10), lipids (method 960.39), and ash (method 920.153). The total carbohydrates content was calculated by difference according to Equation (2):

Carbohydrates (g/100 g) = 100 − (Proteins + Lipids + Moisture + Ashes)(2)

The total caloric content (kcal) of the raw burgers was calculated based on a 100 g sample, considering the energy values for fat (9 kcal/g), protein, and carbohydrates of 4.02 kcal/g [29].

### 2.6. Cooking Losses

The cooking losses were determined using three burger treatments per batch, according to Equation (3) [27]:

(3) $$\%\;\mathrm{Cooking}\;\mathrm{losses}=\frac{\mathrm{Raw}\;\mathrm{weight}\;-\;\mathrm{Cooked}\;\mathrm{weight}}{\mathrm{Raw}\;\mathrm{weight}}\;\times \;100$$

### 2.7. Texture Profile Analysis (TPA)

The TPA was conducted utilizing a TA-HD Plus texture analyzer from Stable Micro Systems in Godalming, UK. The analysis involved employing a 7.5 cm cylindrical plate probe on cylindrical samples measuring 2.5 cm in diameter and 1 cm in height. The samples underwent compression to 50% of their initial height at a consistent speed of 20 cm/min, with both pre-test and post-test speeds set to 20 cm/min [30,31]. The hardness, springiness, cohesiveness, and chewiness were determined [32,33]. 

### 2.8. Lipid Oxidation

The lipid oxidation of the raw burgers stored at −18 °C for eight weeks was determined in triplicate by measuring the thiobarbituric acid-reactive substances (TBARS) using the method Cd 19–90 [34], with the modifications described by Patinho et al. [35]. Seven grams of burger sample was used for the analysis. The TBARS values were calculated by measuring the absorbance at 532 nm using a spectrophotometer (Thermo Scientific, UV-Visible Spectrophotometer, Genesys 150, Madison, WI, USA) and a standard curve (0.6, 1.0, 2.5, 5.0, and 10.0 µM) of 1,1,3,3 tetraethoxypropane and were expressed in mg of malonaldehyde/kg burger sample. 

### 2.9. Sensory Evaluation

#### 2.9.1. Consumers

A panel of 95 regular consumers of burgers was gathered (45% women and 55% men; 17–56 years). These participants presented varying consumption frequencies: 22% of them consumed burgers every 15 days, 5% 1–3 times a week, 44% once a month, and 28% rarely. All the participants signed an informed consent form to participate in this study, which was approved by the Institutional Research Ethics Committee of the Universidad Nacional de la Amazonia Peruana—UNAP (protocol N° PI-006 CIEI-UNAP).

#### 2.9.2. Procedure

To ensure an edible quality, the burgers were subjected to a microbiological analysis of aerobic mesophilic, *Escherichia coli*, *Escherichia coli* O157:H7, *Staphylococcus aureus*, and *Salmonella* sp., according to the regulations of the Sanitary Technical Standard of the General Directorate of Environmental Health of the Ministry of Health of Peru [36].

A Check-All-That-Apply (CATA) sensory test was performed at the Sensory Evaluation Laboratory of UNAP, according to Rios-Mera et al. [37], in a 15 min individual session. The consumers performed the test in individual sensory booths with an artificial white light. The samples (10–15 g) were served monadically and balanced on plates coded with three random numbers following a Williams Latin Square design [38]. The participants rated their overall liking on a nine-point hedonic scale, spanning from “extremely dislike” to “extremely like” [39]. The consumers were instructed to select all the applicable sensory terms to describe the samples (CATA questions). The sensory terms included 16 terms: aromatic, grilled, characteristic, compact, seasoned, hard, off-flavor, fatty, juicy, fishy, spicy, tasty, salty, dry, tender, and non-compact. Those terms were taken from previous research works on fish-based burgers [40]; however, the term non-compact was added after a pre-test due to the effect of MSIO on the texture of the burgers. Water and crackers were offered to the consumers between samples to cleanse their palates.

### 2.10. Fatty Acid Profile

The fatty acid profile of the SIO, fish fillets, and raw burgers (only treatments T1 and T3) was determined by methyl esterification as described by Hartman and Lago [41], with adaptations based on the AOCS [42] Ce 1b-89 method. The lipid samples were extracted (except for the SIO) following the Bligh and Dyer [24] method. The analysis methodology and conditions were taken from Iman et al. [27]. The analysis was conducted within 15 days of burger processing, with measurements being performed in duplicate (two lipid extractions per treatment per batch). The results were expressed as grams of fatty acids per 100 grams of sample.

### 2.11. Data Analysis

The data (except for TBARS, sensory analysis, and fatty acid profile) were analyzed by means of a mixed analysis of variance (ANOVA), considering the treatments as a fixed effect and the batch as a random effect. The TBARS results were analyzed by a factorial design considering the treatments, the storage time (weeks), and their interaction as sources of variation. The overall liking was analyzed by mixed ANOVA, with the treatment as a fixed factor and the subject and presentation order as random factors. Tukey’s test was used for a pairwise comparison at a 5% significance.

For the CATA questions, a contingence table was constructed based on the frequency of the sensory terms used by the consumers to describe each sample [43]. This table was then used to express the sensory profile in a correspondence analysis (CA), considering the chi-square distances [44]. 

The XLSTAT 2015 (Addinsoft, New York, NY, USA, EEUU) and R version 4.3.1 (R Core Team, 2017) software were used for data analysis.

## 3. Results

### 3.1. MSIO Characterization

The EE for MSIO was 80.96%; previously, ref. [45] reported a 75.8% EE when encapsulating SIO with a mixture of AG and maltodextrin (inlet temperature of 150 °C). In a similar study, Nawas et al. [46] reported an 87.50% EE when using AG and Inu as the wall materials for entrapping fish oil (inlet temperature of 170 °C). The different spray-drying conditions between the studies can explain the difference between the results. According to Aghbashlo et al. [47], an increase in the inlet air temperature can produce higher EEs due to rapid crust formation. Likewise, other important variables can affect the EE, such as the drying air mass flow, the air mass flow rate, the feed mass flow rate, and the emulsion droplet size [48]. 

**Figure 1:**
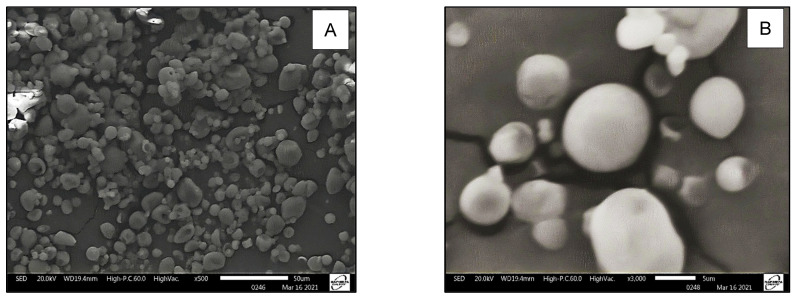
Scanning electron microscopy (SEM) showing micrographs of microencapsulated sacha inchi oil (MSIO). Magnification 500× (**A**) and 3000× (**B**).

The SEM images of MSIO revealed that most of them had spherical and slightly concave shapes with minimum cracks and pores on the surface (Figure 1). These features are desirable in spray-dried microparticles due to the greater protection afforded to the microencapsulated oil [49]. 

To test microencapsulation’s protective effect on SIO, the peroxide value was measured once a week for a month at 45 °C. At time zero, USIO had the lowest peroxide index (2.28 ± 0.76 meq peroxide/kg oil) compared to MSIO (6.44 ± 0.19 meq peroxide/kg oil). Thus, the emulsification and spray-drying processes affected the oxidative stability of SIO. However, at week 1, USIO considerably increased in terms of its peroxide index, to 83.47 ± 6.74 meq peroxide/kg oil. In contrast, MSIO presented a lower value (13.50 ± 1.04 meq peroxide/kg oil). A similar trend was observed in the second (56.20 ± 1.79 meq peroxide/kg oil) and third weeks (90.26 ± 0.86 meq peroxide/kg oil) for MSIO; for USIO, the peroxide values were 111.14 ± 0.96 meq peroxide/kg oil and 118.33 ± 1.39 meq peroxide/kg oil for the second and third week, respectively. However, at week 4, MSIO presented higher peroxide values (151.13 ± 1.26 meq peroxide/kg oil) than USIO (128.36 ± 1.95 meq peroxide/kg oil). 

According to the *Codex Alimentarius* [50], the peroxide value limit for cold-pressed and virgin oils is 15 meq of active oxygen/kg oil, a value which MSIO exceeded in our study after the second week. However, it is important to highlight that the temperature used in our study (45 °C) is not typical for the storage of oils. Thus, high levels of peroxides were expected. Tello et al. [25] also observed increased peroxide values in sunflower oil particles using accelerated oxidation conditions. Still, there was a decrease when the particles were coated with proteins. In this sense, Geranpour et al. [20] cited several strategies to improve the oxidative stability of spray-dried PUFAs, such as the choice of wall materials, the use of natural antioxidants and stabilizers, the production of double-shell and multi-core microcapsules, and control of the storage conditions (adjustments in light, temperature, oxygen, relative humidity, and packaging systems). Applying these strategies could improve the oxidative stability of MSIO and deserves to be evaluated in future studies.

Although the peroxide index used in our research is a valuable indicator of lipid oxidation, especially at the beginning of lipid oxidation [51], it is common to quantify secondary oxidation compounds using TBARS for complex products such as burgers. The application of MSIO in fish-based burgers could impact lipid oxidation, and it indicated the effectiveness of MSIO production under the conditions established in this study.

### 3.2. Proximal Composition and Sodium and Calcium Contents

The proximal composition, cooking losses, and TPA are shown in Table 2. No differences (*p* > 0.05) were observed for the moisture, and the levels were similar to those reported by Saavedra et al. [40] but lower than those found by Presenza et al. [52] in fish burgers. These authors used a higher fish proportion in their burger formulation than in this study, which may have increased the moisture level in their product; furthermore, differences in the protein, lipid, carbohydrates, and ash levels may have been due to differences in the composition of the fish species utilized (Presenza et al. [52] used *Colossoma macroporum*) as well as in the different formulation of the burgers. The lipid content increased significantly in our burgers at higher concentrations of MSIO. This result differs from that reported by Heck et al. [53], who found that microparticles containing chia oil and linseed oil decreased the fat content in beef burgers. These differences could have been due to the characteristics of the microparticles, since those authors used moist microparticles with a high water content (73.3–74.3% of moisture) and we used microparticles in a powder state (3.64 ± 0.17% of moisture). Likewise, the SIO contributed to the increase in ashes in our burgers, but no clear trend was observed in the levels of proteins and carbohydrates after the addition of SIO, which varied from 18.02 to 18.65% and from 5.75 to 7.20%, respectively. On the other hand, the caloric content of the burgers ranged from 267.68 ± 1.04 to 274.61 ± 0.27. These results were higher than those reported by Presenza et al. [52], who reported kcal values between 159.53 and 168.48 for *Colossoma macropomum* burgers with added flours. In another study involving tilapia burgers with chia gels, the caloric content varied from 156.24 to 215.76 kcal [54]. The addition of MSIO to our burgers increased their caloric content, except for T3.

The TPA results show that the hardness and chewiness increased when the levels of MSIO were between 3% and 5%. In other studies involving fish burgers, the addition of flours and starches increased their hardness values [52,55,56]. Likewise, it has been reported that adding microparticles to beef burgers directly impacts their hardness values [37]. Thus, regardless of the meat chosen for the burger formulation, adding flour and starches increases the hardness of burgers. However, in our study, there was a decrease in hardness and chewiness at the highest level of MSIO fortification, indicating that the addition of oil microparticles up to a 6% concentration is not viable in terms of texture since a product with a non-compact structure is obtained (see also the results of sensory characterization). The cooking losses ranged from 32.63 ± 0.54% to 34.16 ± 0.31%, which could be considered irrelevant in practical terms due to the slight numerical difference, indicating that MSIO did not affect this parameter in the burgers.

### 3.3. Lipid Oxidation (TBARS)

**Figure 2:**
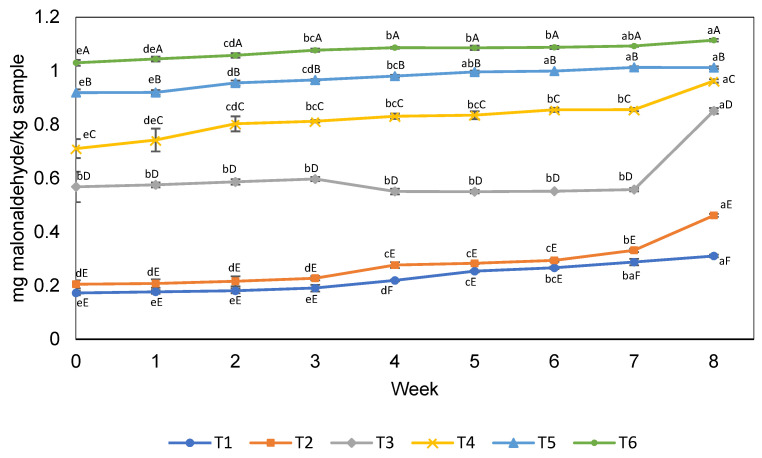
Lipid oxidation of fish-based burgers fortified with unencapsulated and microencapsulated sacha inchi oil. T1 = burger without sacha inchi oil. T2 = 3% unencapsulated sacha inchi oil. T3 = 3% microencapsulated sacha inchi oil. T4 = 4% microencapsulated sacha inchi oil. T5 = 5% microencapsulated sacha inchi oil. T6 = 6% microencapsulated sacha inchi oil. Different letters between the treatments (lower case) in each week and between weeks for the same treatment (upper case) represent a significant difference (*p* < 0.05) between the means obtained with Tukey’s test.

MSIO increased the TBARS values compared to the treatments without SIO and USIO (*p* < 0.05), but all the treatments showed an increasing trend during the eight weeks of storage at −18 °C (Figure 2). The treatment with a higher MSIO (T6) content presented the highest TBARS values, which could be attributed to the higher range of SIO rich in PUFAs. However, SIO microencapsulation also contributed to the increase in TBARS in the burgers. This is evident when the treatments at the same SIO concentration (T2 and T3) are compared, as the lipid oxidation for the MSIO burgers increased from 68% to 177% during storage. Thus, it is important to consider strategies to promote the thermal stability of oils rich in PUFAs in the spray-drying process, as mentioned in Section 3.1 of this paper (MSIO characterization), in which we cited the recommendations of Geranpour et al. [20]. The addition of natural antioxidants can be a solution and is recommended for the application of meat products [57]; likewise, the direct incorporation of antioxidants into the oil encapsulation process can be performed [58]. Heck et al. [53] used this strategy and improved the oxidative stability of beef burgers when using rosemary in the microencapsulation of chia oil. In the context of the development of this study, the Amazon is rich in plant sources with an excellent antioxidant potential; for example, Hanula et al. [59] observed that the incorporation of açaí extract in fat-reduced beef burgers delayed the oxidation determined by TBARS and volatile compounds.

On the other hand, it is necessary to know if the TBARS levels obtained through such processes do not impact negatively the sensory perception of consumers. The ingredients of burgers, such as fish fillets and SIO, may contain high levels of PUFAs, which are very susceptible to oxidation, in which case, the product obtained would not be safe for consumers during the sensory analysis. With this in mind and to avoid excessive lipid oxidation, the antioxidant sodium erythorbate was included in the formulation of the burgers in our study. It is possible that, without sodium erythorbate, the TBARS levels would have been higher, but studying this antioxidant’s level in the burger was not the objective of this study. Therefore, the differences in the TBARS levels between the treatments were directly proportional to the addition of unencapsulated and microencapsulated SIO.

### 3.4. Consumers’ Sensory Characterization and Overall Liking

**Figure 3:**
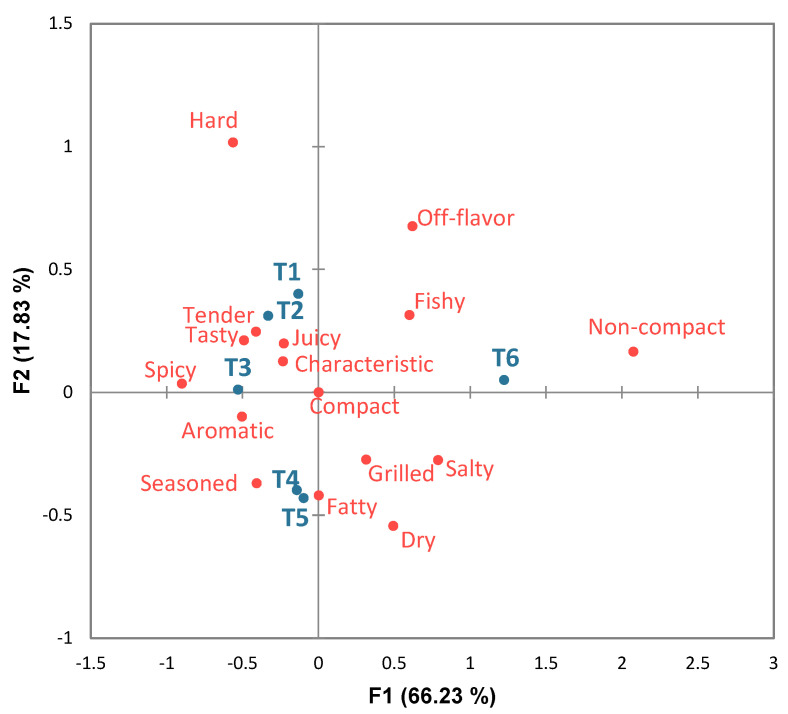
Correspondence analysis (CA) of (a) Check-All-That-Apply (CATA) questions of fish-based burgers fortified with unencapsulated and microencapsulated sacha inchi oil. The treatments are shown in blue and the sensory attributes in red. T1 = burger without sacha inchi oil; T2 = 3% unencapsulated sacha inchi oil; T3 = 3% microencapsulated sacha inchi oil; T4 = 4% microencapsulated sacha inchi oil; T5 = 5% microencapsulated sacha inchi oil; and T6 = 6% microencapsulated sacha inchi oil.

Adding USIO or MSIO into the burgers did not affect the count of microorganisms. Thus, according to Peruvian regulations, the formulated burgers in this study were considered safe for human consumption [36]. The outcomes of CA in the CATA data showed 84.07% of the total variance, with 66.23% and 17.83% for the first and second dimensions, respectively (Figure 3).

Figure 3 shows that the treatments were classified into three groups based on sensory characteristics. In the upper left quadrant, treatments T1, T2, and T3 were characterized mainly by being tender, tasty, juicy, spicy, and characteristic; then, a second group composed of T4 and T5 was reported for the presence of seasoned, fatty, and aromatic attributes. Finally, T6 was characterized by fishy, off-flavor, and non-compact attributes, the latter being related to the lower results in terms of instrumental hardness and chewiness for T6 (Table 2). The presence of fishy and off-flavor sensory attributes could have been related to the oxidation level of T6 caused by the microencapsulation process and the higher amount of SIO added to the burger. The TBARS levels for T6 ranged from 1.03 to 1.11 mg malonaldehyde/kg sample, which could be considered the lipid oxidation threshold detected by the consumers in this study. This aspect has been previously studied and cited by some authors, who reported that the sensory perception threshold of oxidation in meat and meat products is 2 mg malonaldehyde/kg sample [30,60,61]. However, for frozen, chilled, or ice-stored fish, the acceptable values are higher, up to 5 mg malonaldehyde/kg [27].

**Figure 4:**
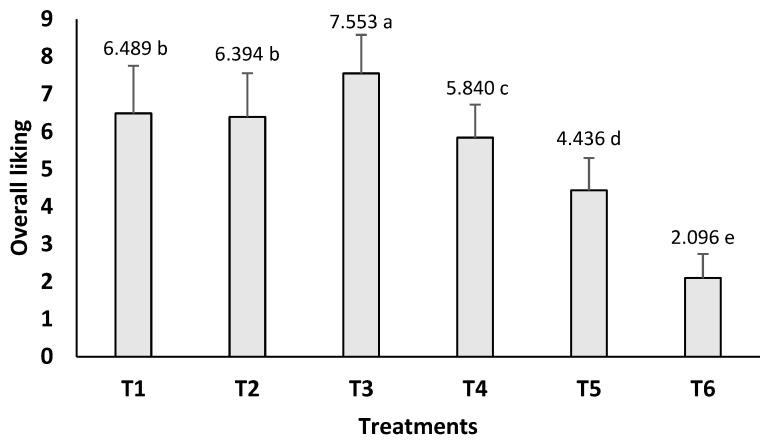
Overall liking of fish-based burgers fortified with unencapsulated and microencapsulated sacha inchi oil. T1 = burger without sacha inchi oil; T2 = 3% unencapsulated sacha inchi oil; T3 = 3% microencapsulated sacha inchi oil; T4 = 4% microencapsulated sacha inchi oil; T5 = 5% microencapsulated sacha inchi oil; and T6 = 6% microencapsulated sacha inchi oil. Different letters on the same barplots represent a significant difference (*p* < 0.05) between the treatments according to Tukey’s test.

On the other hand, in a previous study reported by our group [40], it was observed that the attributes of being tasty, juicy, and tender were positive drivers of consumers liking fish burgers. Conversely, being off-flavor had a negative impact on consumers’ perceptions. In this context, treatment T3, followed by T1 and T2, obtained the highest consumer liking rate (Figure 4). Note that, although the microencapsulation of SIO caused the oxidation of SIO and the burger, this was not enough to compromise the consumers’ acceptance of these burgers; in fact, the highest overall liking score was obtained for treatment T3. Therefore, despite the differences in their proximal composition, instrumental texture, and lipid oxidation, the sensory aspect of the burgers indicated the possibility of fortifying burgers with MSIO up to a 3% concentration without decreasing the overall liking of the product. For this reason, from the nutritional perspective, treatment T3 was subjected to a fatty acid profile analysis, as shown in the following section. In addition, analyses of the fatty acid profiles of the SIO, fish fillets, and burgers without the addition of SIO were conducted to evaluate the effectiveness of MSIO fortification in fish-based burgers.

### 3.5. Fatty Acid Profile

Table 3 shows the fatty acid profile of the SIO, the boquichico fillets, and the raw fish burgers of T1 and T3. The results of the SIO corroborate the works of Chirinos et al. [62] and Vicente at al. [63], who reported that SIO presents a high content of ALA equivalent to 48% to 50%, which, together with linoleic acid, represent above 80% of the PUFAs contained in the oil. Moreover, the ratio n-6/n-3 of SIO was 0.7. For this reason, SIO is a promising alternative to formulate omega-3-fortified foods. 

Regarding the boquichico fillets, palmitic acid (16:0) was the most abundant acid, followed by oleic acid (18:1 n-9). Still, since the fat content of the fillets was low (4.79 ± 0.04% fat), the presence of these fatty acids did not influence the amount of the same types of fatty acids in the burgers. Moreover, the fillets presented a considerable PUFA content, where the sum of EPA and DHA stood out (180 mg). According to MERCOSUR [64], in order for a product to be considered a source of EPA and DHA, it must reach 40–80 mg. Therefore, the fish fillets used in this study could be regarded as food with a high content of long-chain n-3 PUFAs. 

The fortification of the burgers with 3% MSIO (T3) decreased the SFA and MUFA contents. However, it increased the PUFA content. Our results are similar to those of Wongpattananukul et al. [65], where SIO was used as a fat substitute in chicken sausages, decreasing the total saturated fat content. The fortified burger had 640 ± 0.22 mg/100 g of n-3 fatty acids, with ALA being the most predominant n-3 PUFA, an essential fatty acid which the body can use as a precursor of EPA and DHA [66]. Moreover, compared to the burger without SIO (T1), the n-6/n-3 ratio decreased from 7.0 to 3.3. 

To prevent cardiovascular disease, the *Dietary Guidelines for Americans* (2015–2020) suggest the consumption of 450–500 milligrams of n-3 fatty acids per day [67] and an n-6/n-3 ratio of less than 4 [68]. Therefore, the highlighted product in this paper (T3) meets these requirements and can be considered a highly nutritious and acceptable food product. However, SIO’s microencapsulation process must be improved before adding this oil to the product to enhance the oxidative stability of SIO and the burgers it is fortified with. 

Finally, the technological strategy of this study has great potential for industrial applications, with an estimated cost of S/1.90 (PEN) per 100 g of product (approximately USD 0.53), which is relatively cheap for this type of product, due to the availability of the raw materials in the city in which this study was based. In this regard, these burgers could be a healthy eating alternative intended mainly for schools’ feeding programs in Peru, such as Qali Warma.

## 4. Conclusions

The addition of MSIO affected the technological parameters of the fish-based burgers in this study. In fortification levels up to 5%, there was an increase in hardness from 14.48 to 28.07 N and in chewiness from 8.11 to 14.18 N; but, then, there was a drastic decrease in these parameters with 6% MSIO fortification, with levels comparable to the non-fortified burger. As the level of MSIO incorporation increased, there was a corresponding increase in lipid oxidation. The oxidation levels of the MSIO burgers were even higher than those of the USIO burgers, suggesting the need for future studies seeking to improve oxidative stability in microparticle production and during burger processing. Nonetheless, the results show that it is possible to fortify fish burgers with 3% MSIO, with the products of such a treatment presenting positive sensory attributes, such as being *tender*, *tasty*, and *juicy*, and the highest acceptance score, constituting a promising way of fortifying products with ALA. 

## Figures and Tables

**Table 1:** Formulation of fish-based burgers fortified with unencapsulated and microencapsulated sacha inchi oil.

Component (g/100 g)	Treatments					
	T1	T2	T3	T4	T5	T6
Fish fillet ^1^	70	70	70	70	70	70
Pork back fat	20	17	17	16	15	14
USIO ^2^	0	3	0	0	0	0
MSIO ^3^	0	0	3	4	5	6
Salt	1.5	1.5	1.5	1.5	1.5	1.5
Cold water	7	7	7	7	7	7
Monosodium glutamate	0.29	0.29	0.29	0.29	0.29	0.29
Oregano powder	0.2	0.2	0.2	0.2	0.2	0.2
Pepper powder	0.2	0.2	0.2	0.2	0.2	0.2
Onion powder	0.4	0.4	0.4	0.4	0.4	0.4
Garlic powder	0.4	0.4	0.4	0.4	0.4	0.4
Sodium erythorbate	0.01	0.01	0.01	0.01	0.01	0.01

^1^ Boquichico (*Prochilodus nigricans*). ^2^ Unencapsulated sacha inchi oil. ^3^ Microparticles containing sacha inchi oil.

**Table 2:** Mean values and standard deviation of the proximal composition, texture profile analysis, and cooking losses of fish-based burgers fortified with unencapsulated and microencapsulated sacha inchi oil.

Treatments	Proximal Composition (g/100 g)							Instrumental Texture Profile			Cooking Losses (%)
	Moisture	Protein	Lipids	Ash	Carbohydrates	Energy (kcal)	Hardness (N)	Springiness	Cohesiveness	Chewiness (N)	
T1	54.48 ± 0.39 ^a^	18.64 ± 0.17 ^a^	18.43 ± 0.07 ^c^	1.64 ± 0.13 ^b^	6.81 ± 0.49 ^ab^	267.68 ± 1.04 ^b^	14.48 ± 1.25 ^c^	0.84 ± 0.07 ^a^	0.67 ± 0.03 ^ab^	8.11 ± 1.26 ^b^	33.43 ± 0.18 ^ab^
T2	54.43 ± 0.22 ^a^	18.65 ± 0.25 ^a^	18.86 ± 0.06 ^b^	1.88 ± 0.07 ^a^	6.18 ± 0.22 ^bc^	269.06 ± 0.48 ^b^	16.59 ± 1.08 ^b^	0.84 ± 0.02 ^a^	0.69 ± 0.03 ^a^	9.69 ± 0.94 ^b^	33.84 ± 0.10 ^a^
T3	54.30 ± 0.07 ^a^	18.02 ± 0.10 ^b^	18.54 ± 0.21 ^bc^	1.94 ± 0.05 ^a^	7.20 ± 0.36 ^a^	267.72 ± 0.99 ^b^	26.21 ± 1.92 ^a^	0.81 ± 0.07 ^a^	0.63 ± 0.06 ^ab^	13.27 ± 2.35 ^a^	33.49 ± 0.26 ^ab^
T4	54.26 ± 0.22 ^a^	18.33 ± 0.17 ^ba^	18.39 ± 0.05 ^c^	1.89 ± 0.04 ^a^	7.13 ± 0.13 ^ab^	267.33 ± 1.14 ^b^	24.04 ± 2.20 ^a^	0.81 ± 0.06 ^a^	0.62 ± 0.03 ^b^	12.06 ± 1.34 ^a^	34.13 ± 0.35 ^a^
T5	54.38 ± 0.07 ^a^	18.49 ± 0.23 ^ba^	18.84 ± 0.10 ^b^	1.91 ± 0.06 ^a^	6.38 ± 0.15 ^abc^	269.06 ± 0.51 ^b^	28.07 ± 3.25 ^a^	0.82 ± 0.07 ^a^	0.62 ± 0.03 ^b^	14.18 ± 2.17 ^a^	34.16 ± 0.31 ^a^
T6	54.23 ± 0.12 ^a^	18.26 ± 0.17 ^ba^	19.84 ± 0.10 ^a^	1.92 ± 0.07 ^a^	5.75 ± 0.46 ^c^	274.61 ± 0.27 ^a^	14.16 ± 0.78 ^c^	0.80 ± 0.06 ^a^	0.63 ± 0.01 ^ab^	7.16 ± 0.86 ^c^	32.63 ± 0.54 ^b^

T1 = burger without sacha inchi oil; T2 = 3% unencapsulated sacha inchi oil; T3 = 3% microencapsulated sacha inchi oil; T4 = 4% microencapsulated sacha inchi oil; T5 = 5% microencapsulated sacha inchi oil; and T6 = 6% microencapsulated sacha inchi oil. Different letters on the same column represent a significant difference (*p* < 0.05) between the treatments according to Tukey’s test.

**Table 3:** Mean values (g fatty acid/100 g sample) and standard deviation of the fatty acid profile of sacha inchi oil, fish fillets (boquichico, Prochilodus nigricans), and fish-based burgers without sacha inchi oil enrichment (T1) and of those fortified with 3% microencapsulated sacha inchi oil (T3). n.d.: not detected.

Fatty Acid	Sacha Inchi Oil	Fish Fillets	T1	T3
C14:0 Myristic acid	n.d	0.05 ± 0.00	0.22 ± 0.01	0.14 ± 0.01
C16:0 Palmitic acid	3.92 ± 0.114	0.68 ± 0.01	2.87 ± 0.17	2.72 ± 0.04
C16:1 Palmitoleic acid	0.05 ± 0.00	0.10 ± 0.00	0.32 ± 0.02	0.21 ± 0.03
C17:0 Margaric acid	0.10 ± 0.01	0.06 ± 0.00	0.04 ± 0.00	0.06 ± 0.02
C18:0 Stearic acid	2.95 ± 0.04	0.22 ± 0.011	1.59 ± 0.09	1.46 ± 0.06
C18:1 n-9 Oleic acid	8.87 ± 0.259	0.25 ± 0.06	4.86 ± 0.29	3.88 ± 0.18
C18:2 n-6 Linoleic acid	34.26 ± 0.44	0.15 ± 0.00	0.64 ± 0.04	1.99 ± 0.02
C18:2 n-3 α-Linolenic acid	49.28 ± 0.76	0.15 ± 0.00	0.02 ± 0.12	0.46 ± 0.14
C20:0 Arachidic acid	0.16 ± 0.00	n.d.	n.d.	0.03 ± 0.00
C20:1 Eicosenoic acid	0.22 ± 0.04	0.06 ± 0.00	0.04 ± 0.03	0.11 ± 0.00
C20:4 n-6 Arachidonic acid	n.d.	0.10 ± 0.02	0.06 ± 0.00	0.10 ± 0.04
C20:5 n-3 Eicosapentaenoic acid (EPA)	n.d.	0.06 ± 0.01	0.02 ± 0.00	0.05 ± 0.03
C22:6, n-3 Docosahexaenoic acid (DHA)	n.d.	0.12 ± 0.03	0.06 ± 0.01	0.07 ± 0.07
Total SFA	7.10 ± 0.06	1.05 ± 0.02	4.72 ± 1.33	4.46 ± 0.07
Total MUFA	9.11 ± 0.32	0.51 ± 0.01	5.22 ± 2.71	4.26 ± 0.13
Total PUFA	83.57 ± 0.30	0.69 ± 0.05	0.80 ± 0.30	2.86 ± 0.28
Total n-3	49.28 ± 0.76	0.38 ± 0.03	0.10 ± 0.26	0.64 ± 0.22
Total n-6	34.26 ± 0.44	0.29 ± 0.03	0.70 ± 0.42	2.13 ± 0.06
Total n-9	8.87 ± 0.26	0.33 ± 00.1	4.83 ± 0.29	3.91 ± 016
n-6/n-3 ratio	0.70	0.80	7.00	3.33

## Data Availability

The original contributions presented in the study are included in the article, further inquiries can be directed to the corresponding author.
